# Endoscopic-Assisted Evacuation: A Useful Adjunct for the Management of Prepatellar Hematomas

**DOI:** 10.7759/cureus.65718

**Published:** 2024-07-30

**Authors:** Srinivas B. S Kambhampati, Mounika N Chodavarapu, Sravya T Paleti, Anirudh P. S Kambhampati, Riccardo D'Ambrosi

**Affiliations:** 1 Orthopaedics, Sri Dhaatri Orthopaedic, Maternity & Gynaecology Center, Vijayawada, IND; 2 Orthopaedics, Siddhartha Medical College, Vijayawada, IND; 3 Orthopaedics, Alluri Sitarama Raju Academy of Medical Sciences, Eluru, IND; 4 Orthopaedic Surgery, Scientific Institute for Research, Hospitalization and Healthcare (IRCCS) Galeazzi Orthopedic Institute, Milan, ITA; 5 Biomedical Sciences for Health, Università degli Studi di Milano, Milan, ITA

**Keywords:** endoscopic-assisted evacuation, orthopedic sports medicine, patella surgery, knee, prepatellar hematoma

## Abstract

Management of prepatellar hematomas can be difficult, and recurrences or persistent oozing are common. Endoscopic-assisted management of these hematomas has not been reported. We present the case of a 44-year-old lady with traumatic prepatellar hematoma who was managed with the evacuation of the hematoma assisted by endoscopic visualization and debridement. She recovered completely within three weeks and remained asymptomatic on the last follow-up at six months. Endoscopic visualization of the hematoma cavity is a useful adjunct to managing prepatellar hematomas, with the potential to identify and control sources of future concerns in wound healing.

## Introduction

Prepatellar hematomas, although relatively rare, pose significant challenges in terms of diagnosis and management. These hematomas are commonly precipitated by trauma, anticoagulant therapy, Morel-Lavallee lesions, and coagulation disorders, with patients on therapeutic anticoagulation facing a heightened risk. The complexity in managing prepatellar hematomas arises not only from their tendency to recur and cause persistent oozing [1] but also from the difficulty in effectively diagnosing them due to their deep subcutaneous location and varied presentation.

The condition typically manifests as a localized swelling over the kneecap, which can be exacerbated by continuous activity or additional trauma, leading to increased pressure in the dermal and subdermal capillaries. This elevated pressure can result in necrosis of substantial areas of the overlying skin, further complicating the clinical scenario [2]. Despite its impact, the literature on the effective management of prepatellar hematomas is sparse, primarily comprising case reports and small case series, reflecting the need for more robust data and innovative treatment approaches.

Historically, the management of prepatellar hematomas has involved conservative measures such as compression, icing, and elevation, alongside more invasive procedures such as aspiration and open surgical evacuation. However, these methods often do not address the potential for recurrence or complications arising from internal bleeding and other underlying vascular issues. The advent of endoscopic techniques in the management of similar conditions, such as septic bursae, hints at potential advancements in treating prepatellar hematomas. Yet, reports of endoscopy-assisted management, although present for septic etiology, are difficult to find for aseptic pathology in the existing literature.

Our case report introduces a novel approach to the management of prepatellar hematomas, employing mini-incision evacuation assisted by endoscopic visualization, which allows for more precise and controlled removal of the hematoma while potentially mitigating the risks associated with traditional surgical methods. This technique not only addresses the immediate physical manifestations of the hematoma but also offers a unique vantage point for identifying and treating sources of future complications, thereby broadening the scope for improved patient outcomes in cases of complex prepatellar hematomas. We report a case of prepatellar hematoma managed by mini-incision evacuation assisted by endoscopic visualization.

## Case presentation

A 44-year-old lady presented as an outpatient with a history of a fall at home followed by swelling of her left knee. Aspiration of blood was performed a week after the injury by a local doctor. The swelling subsided but recurred in three days. She presented to us five days after the aspiration. Her past history included hypothyroidism, for which she was on 125 µm thyroxine for two years, and right-sided renal calculus.

**Figure 1:**
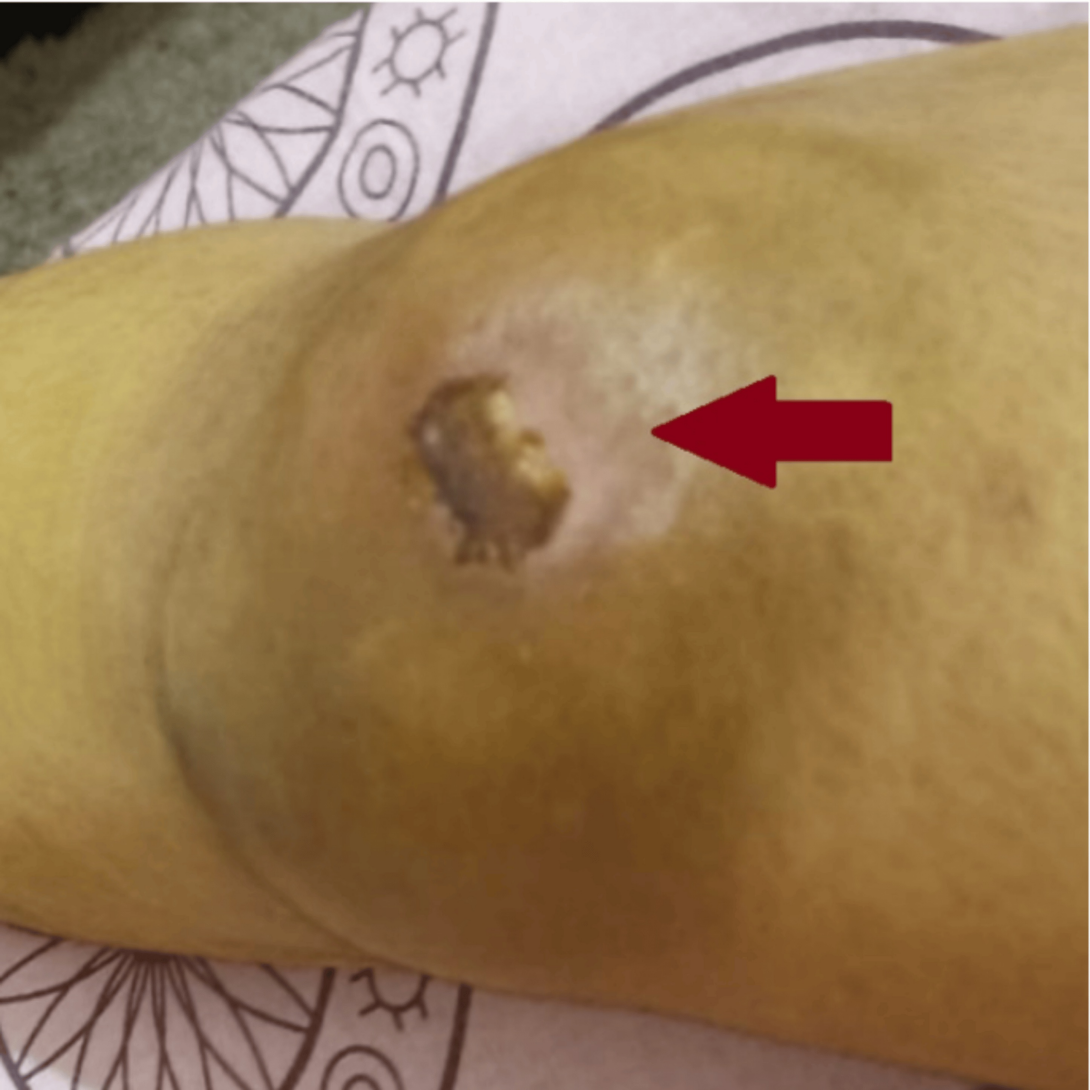
Preoperative swelling and bruising of the overlying skin (red arrow) along with a healing superficial abrasion.

On clinical examination, she had a prepatellar hematoma, with her knee appearing stable. There was grade 1 knee effusion with a flexion range of movement limited to 90 degrees due to pain. A large 15 x 10 cm prepatellar collection with bruising of the overlying skin was noted, along with a healing superficial abrasion superomedially. No active ooze was seen. The collection appeared superficial to the patella (Figure 1).

**Figure 2:**
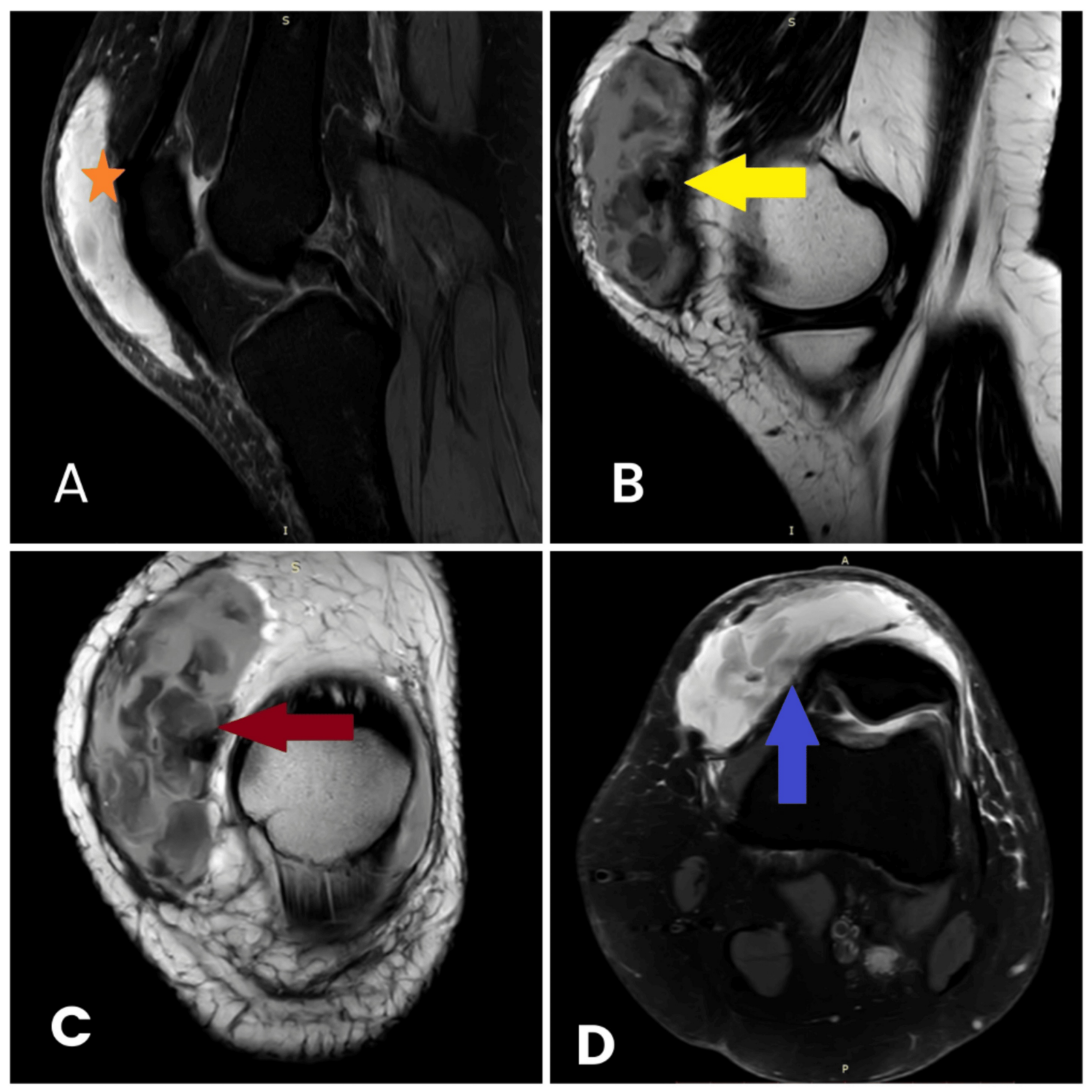
Preoperative MRI of the right knee. A: Sagittal section with prepatellar fluid collection (*). B: Sagittal section displays the extent of the prepatellar collection (yellow arrow). C: Coronal section with the red arrow pointing to the collection. D: Transverse section with the blue arrow pointing to the collection.

An MRI of the knee showed a large prepatellar hematoma (Figure 2). No intra-articular injuries were found. There was some edema of the anterior cruciate ligament.

**Figure 3:**
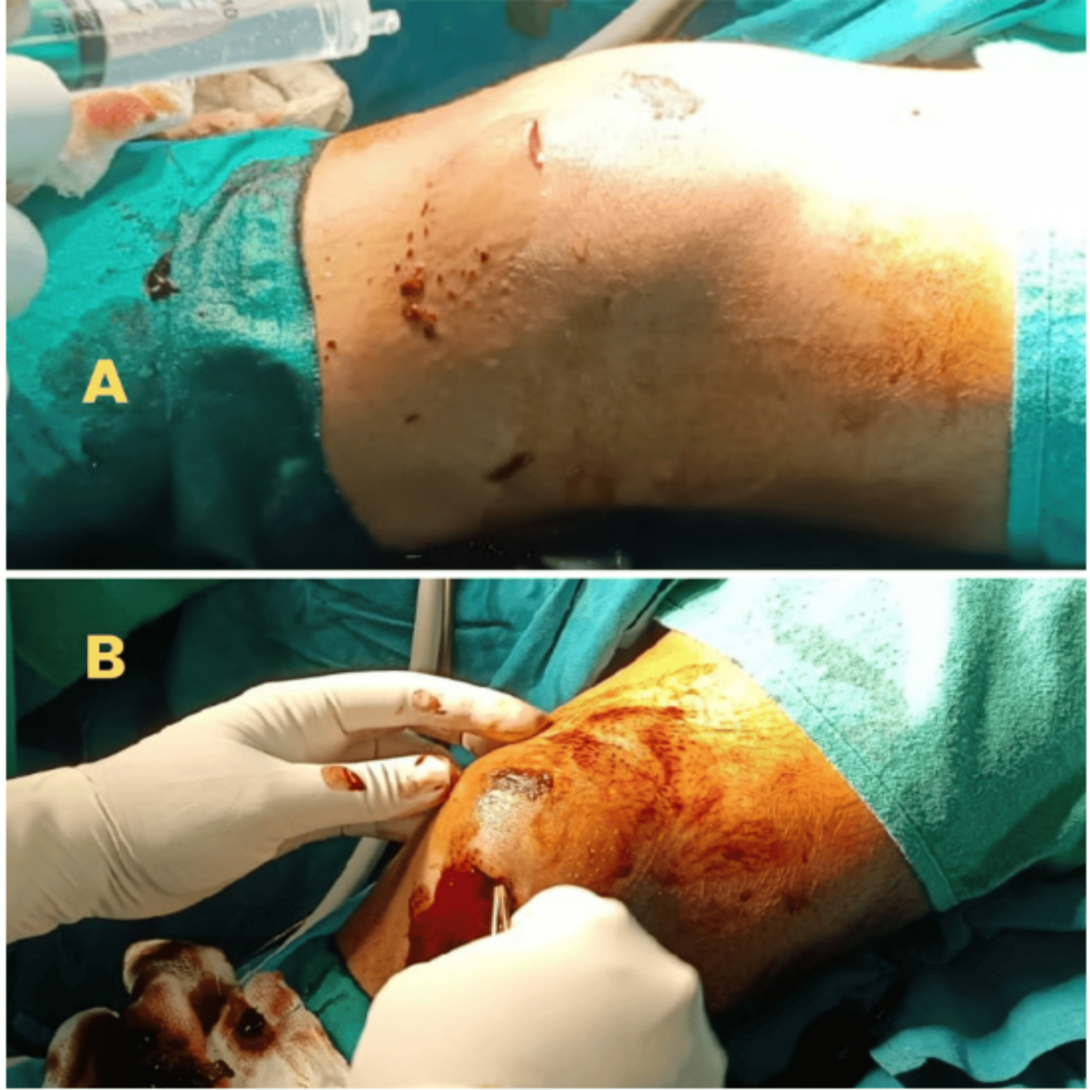
Intraoperative images. A: During flushing of the space with normal saline. B: Evacuation of the clots.

Under spinal anesthesia and a high thigh tourniquet, an incision of about 2 cm was placed at the inferior margin of the swelling. A curette was introduced to evacuate all blood clots. The space was flushed with saline to wash the contents until clear fluid returned (Figure 3).

**Figure 4:**
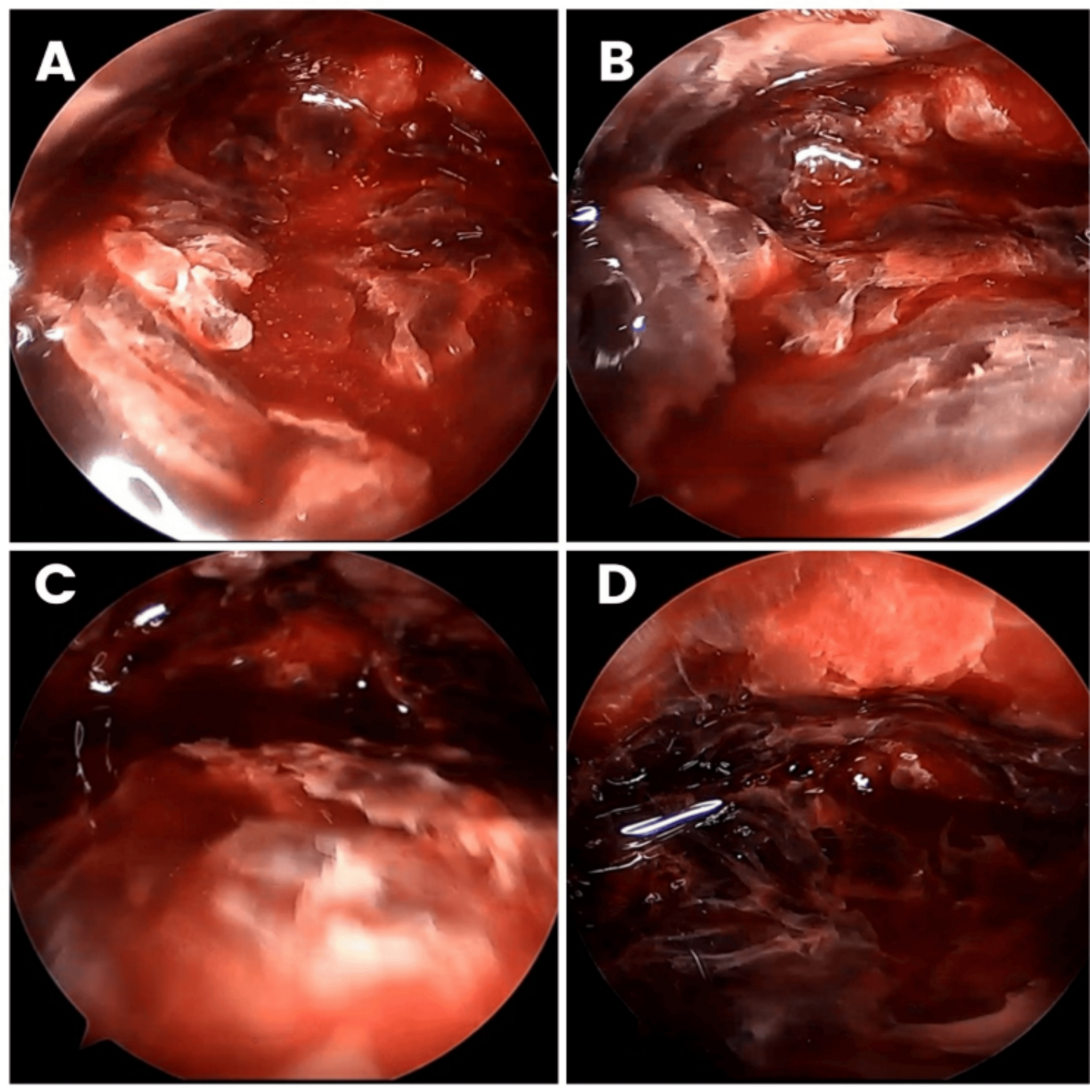
Dry endoscopic images of the evacuation of prepatellar hematoma of the right knee. A, B: After clearing the residual clots. C, D: Before clearing the clots.

An endoscope (standard arthroscope) was then introduced to perform a dry endoscopy (without gas or irrigation fluid) to inspect the cavity. Any residual clots found were removed using a curette or shaver through the same incision, and the cavity was flushed (Figure 4).

**Figure 5:**
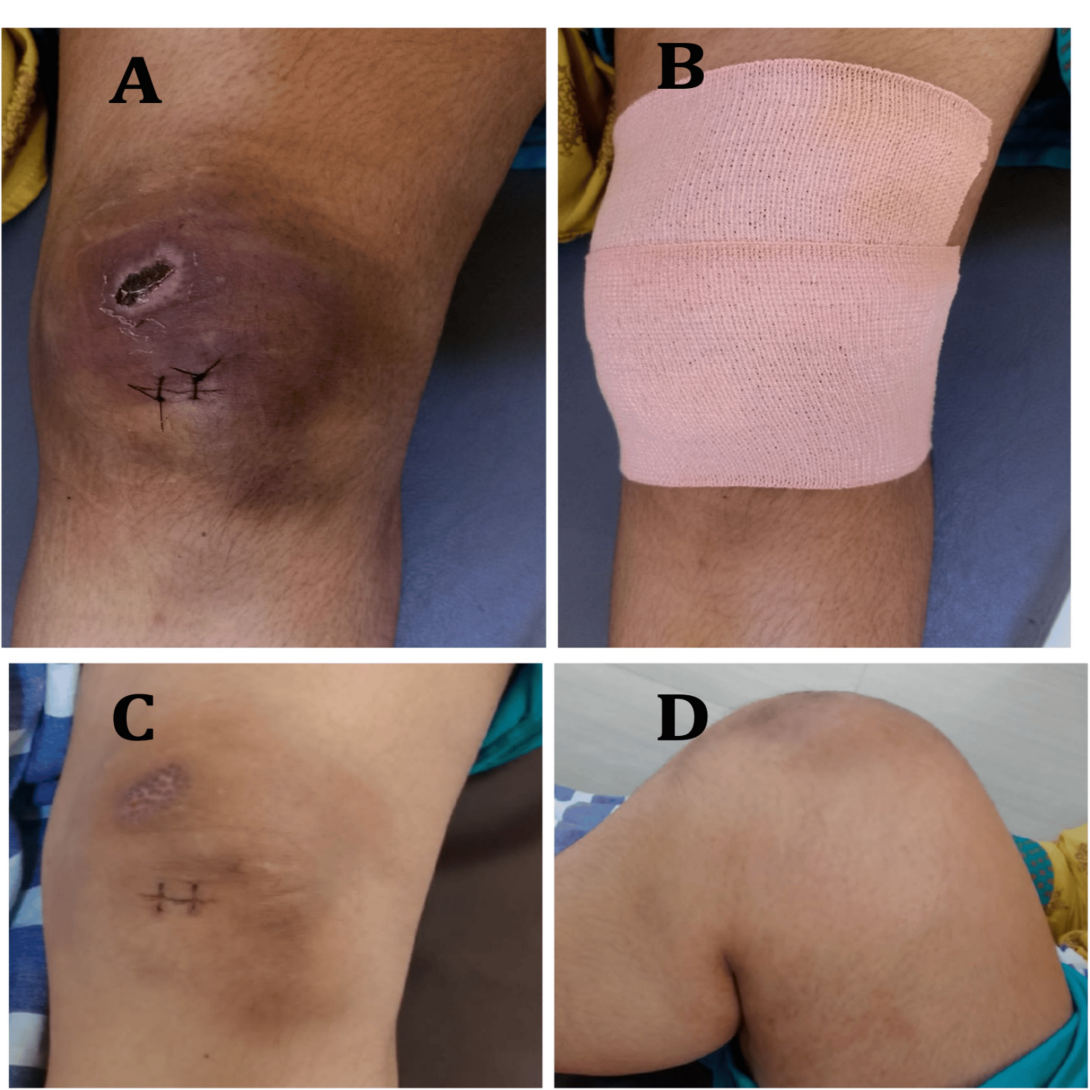
Postoperative clinical images. A: Sutured wound. B: Elastoplast dressing of the wound. C: Healed surgical and abrasion scars following suture removal. D: Full flexion of the knee at three weeks.

The tourniquet was then deflated to inspect for bleeds. We did not find any bleeders in our case. The cavity was then decompressed, and the wound was closed. Dressing was done using an Elastoplast cover to maintain pressure and compress the cavity to prevent a recollection of blood (Figure 5).

The patient was discharged on day one postoperatively. She became asymptomatic three weeks after surgery and returned to normal activities. On the last follow-up six months after surgery, she was completely asymptomatic.

## Discussion

The important message of this report is that endoscopy is a useful adjunct to the management of prepatellar hematomas and, coupled with elastic dressings postoperatively, can reduce the chances of recurrences or oozing. Prepatellar hematoma is a rare condition, with only a few cases reported in the literature [1]. While pretibial hematomas are relatively common in elderly populations on anticoagulants, prepatellar hematomas are rare and reported as individual case reports in the literature. Causes include trauma, hemorrhagic bursitis, and Morel-Lavallee lesions.

The anatomy of the prepatellar bursal area consists of potential spaces where a hematoma can collect after trauma [1]. There is also an extensive prepatellar vascular anastomotic network over the anterior surface of the patella [3] that could be the source of the hematoma following trauma, which can potentially cause recurrences and delayed healing. Even varicose veins have been implicated with persistent oozing from the wound after drainage [1]. Due to the above factors, hematomas in this region are at a higher risk of recurrence or persistent ooze. If the inside of the hematoma can be visualized and active bleeders identified and coagulated, recurrences can be reduced. The hematomas are a combination of venous and/or arterial bleeding, depending on the cause. Direct trauma or a shearing force leading to Morel-Lavallee lesions are common causes in young patients.

Management of prepatellar hematomas reported in the literature includes open evacuation only. The problem with this approach is that small bleeders inside the cavity are not visualized, especially in a Morel-Lavallee-type lesion, leading to recurrences. Apart from a risk of sepsis due to the hematoma, recurrences have been reported in the literature. Even our case had a recurrence of hematoma after initial aspiration. These are not amenable to aspiration after the hematoma coagulates to a clot. While some may argue the swelling may be evacuated under local anesthesia, due to the size of the lesion and recurrence and the possibility of finding a bleeder inside, we opted to operate under spinal anesthesia with endoscopic visualization as an adjunct.

Hemorrhagic bursae occur deep into the subcutaneous plane and are limited by the capsule of the bursa. The type of collection gives a clue to the underlying condition [1]. While dark venous fluid clots are seen from venous injury, reddish clots are seen for arterial injury, and a combination of blood with or without clots and fluid is seen with hemorrhagic bursitis. Our case was straightforward, with a clear history of trauma.

To prevent recurrences, it is important to visualize the inside for active bleeders. Hence, we first evacuated the collection using a small incision, and washing until clear fluid returned. We introduced the endoscope to visualize the inside and found some residual blood clots that could be the source of infection, persistent ooze, or a hidden bleeding vessel. We evacuated those clots using a wash and a shaver through the same incision. Alternatively, the shaver may use another portal to triangulate and remove the clots. Once satisfied with the clot evacuation, we deflated the tourniquet and confirmed no active bleeding under vision. If any are found, the co-ablator may be used to stop any active bleeders. A cautery may even be used in dry endoscopy with an appropriate instrument through another portal to control oozing. The advantages and disadvantages of endoscopic resection of prepatellar bursa have been summarized in Table 1.

**Table 1:** Advantages and disadvantages of endoscopic resection of prepatellar bursa.

Advantages	Disadvantages
Minimally invasive procedure	Requires a skillful arthroscopist if no radiofrequency device is available
Can be performed under local/regional anesthesia	Hemostasis can be difficult without a radiofrequency device
Reduces tegumentary complications such as infection, wound breakdown, or necrosis of the skin	Difficulty in visualization if blood ooze mixes with irrigation fluid. Hence, the tourniquet is released after evacuation is done
Allows complete removal of the hematoma under visualization	
Prevents future complications of oozing or recurrences	
Useful technique for patients on anticoagulants	

An issue with Morel-Lavallee lesions is that the blood supply to the skin is precarious, and extensive skin incisions compromise cutaneous blood supply and wound healing. In such cases, multiple aspirations would be required along with sclerosants to avoid recollection in the potential space [4]. Using an endoscopic approach, the skin would not be threatened, and sources for oozing and recurrence could be identified and dealt with during the first surgery. Irrigation may be used to inspect the cavity, but the fluid could mix with blood and cause difficulty in visualization. A dry scope (without using gas or fluid) is a useful method for inspecting while opening the cavity using a retractor through another portal to prevent the collapse of the cavity while working in it. Another critical step is to use an Elastoplast or an elastic dressing over the wound to compress the prepatellar area to prevent further collections.

Our technique can be particularly helpful in cases of Morel-Lavallee lesions, where extensive incisions may put the skin at risk and the hematoma cavity may need to be inspected to control oozing/bleeding.

The disadvantage of ultrasound-guided procedure is that bleeding vessels are hard to identify and control. Although the hematoma can be delineated well with ultrasound, its management may be challenging in the presence of bleeders. We did not include ultrasound-guided evacuation in the discussion as it does not offer major advantages in a subcutaneous hematoma as the hematoma is readily accessible.

On the other hand, arthroscopic visualization not only gives visual access to the hematoma cavity it also has the advantage of identifying and controlling any bleeding vessel inside the cavity and ensures complete removal of blood clots from inside the cavity. Any leftover blood clots can cause residual oozing from the wound in a blind procedure [5].

## Conclusions

The integration of endoscopic visualization in the management of prepatellar hematomas, as demonstrated in this case, highlights its utility in enhancing visual access to the treatment area and improving outcomes by reducing the risk of complications such as recurrences and persistent oozing. This approach allows for a detailed inspection of the hematoma cavity, enabling the precise identification and treatment of potential bleeding sources that are otherwise difficult to manage through traditional methods. Our case confirms that endoscopic-assisted evacuation can be a pivotal technique in managing complex cases where conventional treatments might fail to provide satisfactory results. The success noted in this patient, who remained asymptomatic six months post-operation, underscores the potential of this technique to provide lasting relief and prevent future complications.

Furthermore, applying this technique in clinical practice could revolutionize the management strategies for hematomas, particularly in scenarios where patients are at an increased risk due to underlying conditions such as coagulation disorders, Morel-Lavallee lesions, or therapeutic anticoagulation. The ability to visually guide the debridement process not only minimizes invasive procedures but also enhances the overall safety and efficacy of the treatment. Our findings advocate for further studies and consideration of endoscopic visualization as a standard adjunct in hematoma management protocols, aiming to establish a more definitive treatment pathway that could potentially reduce healthcare burdens associated with this condition.
